# Guardians of the epithelium: macrophages protect against toxic fungal derivatives

**DOI:** 10.1038/s41385-020-00369-0

**Published:** 2021-01-25

**Authors:** Allan M. Mowat, Calum C. Bain

**Affiliations:** 1 Centre for Immunobiology, Institute of Infection, Immunity and Inflammation, University of Glasgow, Glasgow, G12 8TA UK; 2 University of Edinburgh Centre for Inflammation Research, Queens Medical Research Institute, Edinburgh, EH16 4TJ UK

## Abstract

A recent paper in *Cell* proposes a new role for macrophages in the distal colonic mucosa, namely the generation of balloon-like processes (BLPs) that sample luminal contents and protect epithelial cells from the toxic effects of fungal metabolites absorbed during this process. Here Allan Mowat and Calum Bain discuss the implications of these novel findings for intestinal physiology and macrophage biology, highlighting how they extend our understanding of how tissue resident macrophages can adapt precisely to the physiological needs of individual anatomical niches.

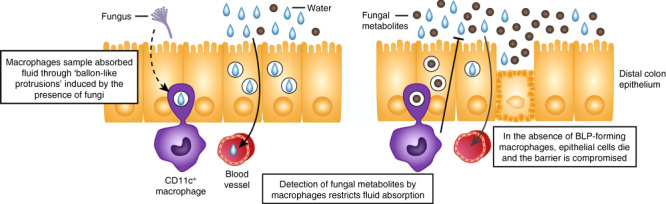

Macrophages are one of the most abundant immune cells in the normal intestinal mucosa.^1^ Given their proximity to the epithelium and their phagocytic properties, it has always been assumed that the major role of mucosal macrophages in vivo would be the capture and destruction of microbes that had penetrated the epithelial barrier from the lumen. In this way, they would play an important part in the body’s mutualistic relationship with the microbiota and in defence against enteric pathogens.^1,2^ While this may indeed be the case, increasing evidence from other organs shows that resident macrophages have crucial physiological roles in maintaining tissue integrity rather than just immune defence or promoting inflammation. Such effects often involve intimate, two-way interactions with tissue cells and amongst others, they include promotion of nerve cell growth and function, cardiac muscle function, maintenance of the healthy alveolar space in the lung and regulation of hair follicle growth.^3^ In the intestine, it has been suggested that resident macrophages of the submucosa and muscularis control smooth muscle activity and hence peristalsis,^4,5^ while there is also evidence that macrophages in the region of the crypts may regulate epithelial stem cell function via production of trophic mediators and/or by influencing Paneth cell behaviour.^1^

The paper by Chikina et al. now adds a novel physiological role for colonic macrophages by demonstrating their ability to sample the fluid material taken up from the lumen by epithelial cells in the distal colon and by detecting the presence of fungal toxins, the macrophages can protect the epithelial cells from damage.^6^

The authors begin by showing that depletion of tissue macrophages by administration of diphtheria toxin (DT) to mice expressing the human DTR under control of the macrophage-specific CD64 (*Fcgr1*) promoter leads to apoptosis of epithelial cells in the distal colon, but not in more proximal segments of the intestine. This effect was associated with increased uptake of hypotonic fluid from the lumen and was replicated by in vivo treatment with an antibody against the macrophage growth factor, CSF1, confirming the specific role of macrophages in the phenomenon. The difference in macrophage behaviour between the distal and proximal colon correlated with the presence of “balloon-like protrusions” (BLPs) that were formed by macrophages immediately underlying the crypt epithelium and which were found within the epithelial layer in intimate contact with the basal surface of epithelial cells. Elegant fluorescence microscopy showed that these BLPs contain large numbers of vesicles of lysosomal/late endosomal origin that contain class II MHC, invariant chain (Ii—CD74) and membrane derived from surrounding epithelial cells. These BLPs are very different in appearance from so-called ‘trans-epithelial dendrites’ that have been suggested to emanate from macrophages or dendritic cells in other parts of the intestine^7^ and unlike them, the BLPs do not cross the epithelial layer and do not enter the lumen itself. However they were shown to take up material from luminal fluid and indeed the number of BLPs increased when colonic fluid uptake was stimulated by intrarectal administration of hypotonic saline or the hormone aldosterone. By using single cell RNA sequencing of F4/80^+^ macrophages from the proximal and distal colon, the authors revealed an enrichment of CD11c^+^ expressing macrophages in the latter, and these aligned transcriptionally with macrophages described as being epithelial-associated in a previous study.^8^ Interestingly, the scRNAseq analysis did not reveal heterogeneity within the total CD11c^+^ macrophage compartment, suggesting that BLP-forming macrophages do not form a transcriptionally-distinct subset of macrophages, but that all CD11c^+^ macrophages might be able to perform this function.

As the major physiological role of epithelial cells in the distal colon is to reabsorb water from the lumen, these results are consistent with the idea that the local macrophage population regulates this homoeostatic function. However the authors extend their findings by providing evidence that this behaviour may be particularly adapted to sampling and responding to metabolites of fungal origin. Thus the number of macrophage-derived BLPs was markedly reduced by treatment of mice with anti-fungal drugs and these agents also prevented the epithelial cell apoptosis seen in macrophage deficient CD64-DTR mice. In parallel, lower numbers of BLPs were found in germ free (GF) mice and these were restored by mono-colonisation with the fungus *Candida albicans*. Interestingly, and in complete contrast, conventional antibiotics did not affect BLPs and the bacterial species present in Schaedler’s altered flora could not restore BLPs in GF animals. A further important finding was that these effects were selective to BLP formation, as total macrophage numbers were not influenced by the presence of fungi, nor was the ability of macrophages to send thinner, more dendritic processes into the epithelial layer, a phenomenon which occurred in both distal and proximal colon.

The authors then attempted to define how the macrophage BLPs might recognise fungi. Somewhat surprisingly, this did not involve the Dectin-1 surface receptor which macrophages and other innate immune cells use to recognise fungi via their cell wall glucans, as BLP formation was normal in mice lacking Dectin-1. In contrast, the reduced numbers of BLPs found in anti-fungal treated mice could be restored by luminal administration of gliotoxin, a toxin produced by a number of fungal species that may include *C albicans*.^9^ Gliotoxin also exacerbated the colonic epithelial cell apoptosis found in CD64-DTR mice, suggesting that this phenomenon might reflect the loss of a macrophage-dependent protective effect on epithelial cells that have taken up the toxin. This conclusion was further supported by the finding that in wild type animals, epithelial cell uptake of gliotoxin-containing fluid from the lumen terminated within 5 min, but this did not occur in the absence of macrophages, exposing the epithelial cells to sustained effects of the toxin. Finally, mice lacking the invariant chain (CD74) phenocopied macrophage deficient mice in terms of the induction of BLPs and apoptosis after administration of gliotoxin, indicating that uptake of exogenous material into endosomal vesicles was involved in the function of macrophages.

This intriguing study reveals a novel and unsuspected role for macrophages in the colonic mucosa, that of forming BLPs which are in close contact with neighbouring epithelial cells, allowing them to sample fluid taken into the epithelial cells and detect the presence of harmful contents. Under these circumstances, the macrophages instruct the epithelial cells to halt uptake of fluid and protect them from death, thus maintaining barrier integrity. As outlined by the authors, the presence of BLPs only in the distal colon is entirely consistent with the primary physiological function of this tissue being water absorption which needs to be fulfilled in the presence of a dense microbiota that includes fungi capable of producing cytotoxins. A water-impermeable epithelial barrier is crucial for this process to continue and this is dependent on highly regulated transcellular routes of fluid uptake such as Aquaporin.^10^ The authors propose that mucosal macrophages quality check these processes and act as gatekeepers by downregulating water uptake channel function in the presence of danger. However, because of the very tight nature of the epithelial barrier in this tissue, the macrophages themselves cannot enter the epithelium to sample the lumen directly and avoid this problem by the formation of flexible BLPs that allow fluid uptake into endosomal vesicles. BLPs are not a feature of other segments of the intestine, where water absorption is not a primary concern, the microbiota is less dense and the looser nature of the barrier might allow macrophages to extend processes all the way through the epithelial layer into the lumen.

Together, the findings add to the ever growing range of physiological roles which resident macrophages can fulfil and underline the exquisite way in which these cells can adapt to highly specific anatomical niches.^11^ Among the intriguing issues that remain to be addressed include whether fungi are indeed the only organisms that stimulate the formation of protective BLPs and the range of the organisms involved remains to be determined. How the macrophages detect the presence of toxic material taken into the BLPs is also unclear and the cellular machinery that specifically allows CD11c^+^ macrophages in the distal colon to form BLP is unclear. It will also be important to establish how the macrophages then inhibit fluid uptake and protect epithelial cells. The authors cite one potential mechanism being inhibition of Aquaporin by PGE_2_ and the role of this pathway and others is something that should be addressable by techniques such as single cell RNAseq analysis of cell populations isolated from individual segments of the intestine. Finally, it remains to be established whether BLPs and macrophage-dependent sampling of fungal products occurs in the human intestine.
